# Esophageal bezoar formation following enteral nutrition

**DOI:** 10.1007/s12328-026-02284-6

**Published:** 2026-02-11

**Authors:** Xueni Liu, Masaya Iwamuro, Chihiro Sakaguchi, Shoichiro Hirata, Katsunori Matsueda, Yuji Kurata, Tatsuhiko Shimizu, Motoyuki Otsuka

**Affiliations:** 1 Department of Gastroenterology and Hepatology, Okayama University Graduate School of Medicine, Dentistry, and Pharmaceutical Sciences, Okayama, 700-8558 Japan; 2 Department of Cardiovascular Surgery, Okayama University Hospital, Okayama, 700-8558 Japan; 3 Department of Anesthesiology and Resuscitology, Okayama University Graduate School of Medicine, Dentistry, and Pharmaceutical Sciences, Okayama, 700-8558 Japan

**Keywords:** Casein, Enteral nutrition, Esophageal bezoar, Nasogastric tube, Postoperative complication

## Abstract

Esophageal bezoars caused by enteral nutrition are rare but may occur when casein-containing formulas solidify under conditions of impaired esophageal clearance. We report a case of complete esophageal obstruction associated with short-term postoperative enteral feeding. A 27-year-old man developed dysphagia and nasogastric tube obstruction after 5 days of nasogastric administration of a casein-based enteral formula following cardiothoracic surgery. Upper endoscopy revealed a large, whitish bezoar completely filling the esophageal lumen. Partial endoscopic fragmentation was achieved, and most of the mass was successfully dislodged into the stomach, enabling reinsertion of the feeding tube. The patient recovered uneventfully, and no recurrence was observed after switching to a peptide-based formula. To explore potential contributing factors, an in vitro experiment was conducted to assess the behavior of the casein-based formula under acidic conditions. The formula remained liquid when mixed with solutions at pH 2.0–5.5 but rapidly solidified under extreme acidic conditions (pH 1.0). In contrast, rapid solidification occurred upon mixing with a pH 1.0 solution. These findings suggest that casein-containing formulas may undergo coagulation under certain physicochemical conditions; however, bezoar formation in this case was likely multifactorial, involving transient postoperative esophageal stasis, impaired clearance, and formula retention rather than sustained exposure to highly acidic gastric juice alone. This case also highlights that even transient postoperative esophageal stasis can precipitate bezoar formation when casein-containing enteral nutrition is administered.

## Introduction

Esophageal bezoars are rare, typically associated with underlying esophageal motility disorders, structural abnormalities, and postoperative anatomical alterations [1, 2]. Although most reported bezoars comprise food residues, medications, or foreign materials, those from enteral feeding formulas are rare. Enteral nutrition is widely used in critically ill or postoperative patients; however, impaired esophageal transit, formula reflux, or prolonged retention may lead to coagulation and solidification of nutritional products within the esophageal lumen [3–6]. Although several case reports have described bezoars from casein- or peptide-based enteral formulations, the clinical characteristics and optimal management of such bezoars remain unclear. Here, we report a rare case of an esophageal bezoar from retained enteral nutrition after cardiothoracic surgery, successfully treated with endoscopic removal.

## Case presentation

**Fig. 1:**
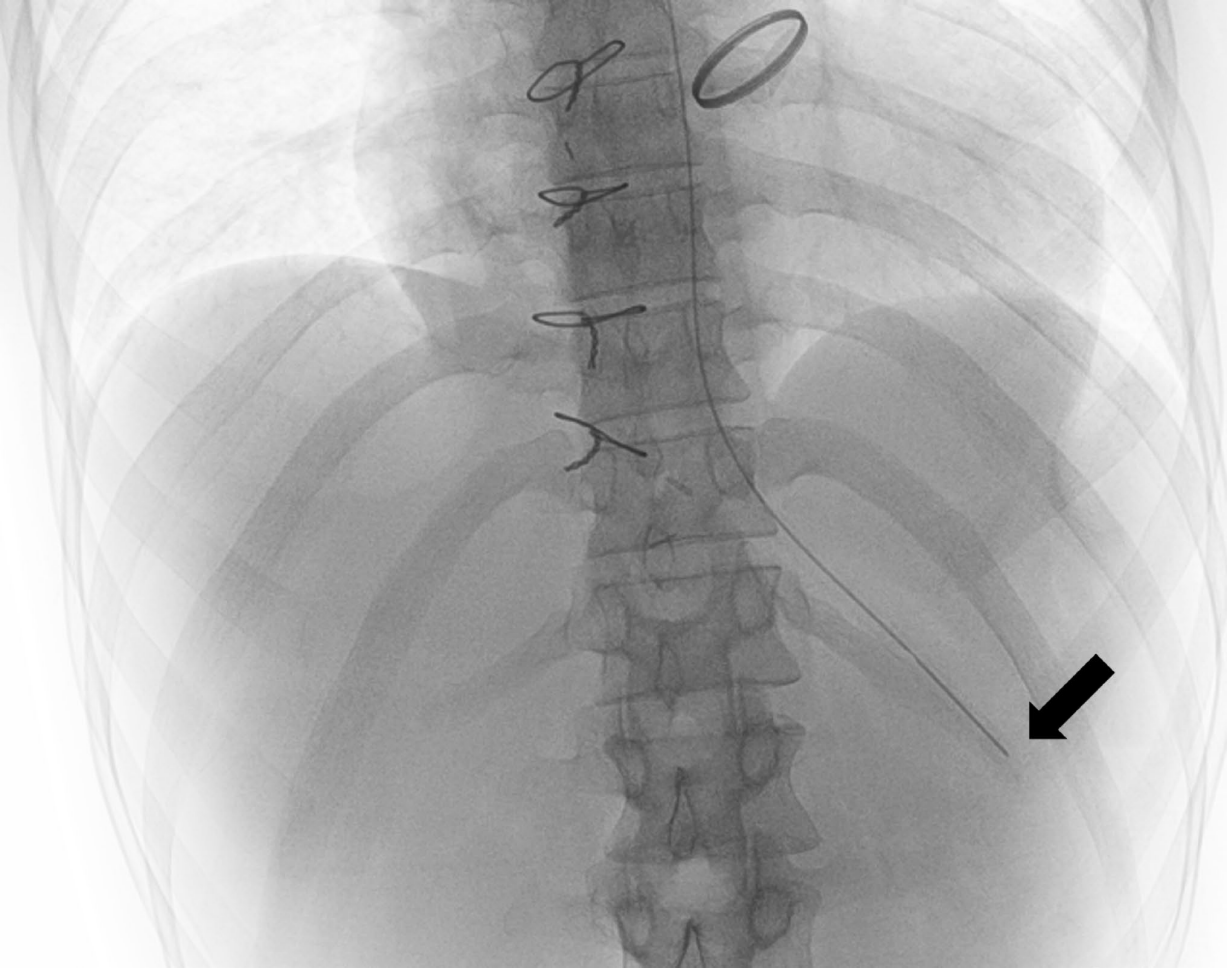
Chest radiograph demonstrating correct intragastric positioning of the nasogastric tube. The tube tip is located within the stomach (arrow), confirming appropriate placement prior to the initiation of enteral nutrition

A 27-year-old man with a history of transposition of the great arteries (type I) and bicuspid pulmonary aortic valve underwent aortic valve replacement with a 27-mm St. Jude Medical mechanical valve, left pulmonary artery reconstruction, and pulmonary artery reconstruction using a 13-mm J-graft 4 years previously. Presently, the patient underwent repeat pulmonary artery reconstruction. On the day of surgery, the patient experienced decreased oxygenation post-extubation, requiring high-flow nasal cannula oxygen therapy. Chest radiography revealed right hemidiaphragm elevation. Because of persistent hypoxemia, the patient was reintubated on postoperative day (POD) 1. A nasogastric tube was placed on POD 3, and correct intragastric positioning of the tube tip was confirmed by radiography (Fig. 1, arrow), after which enteral nutrition was initiated using the Termeal® series nutrition formula (Nutri Co., Japan). On POD 8, the nasogastric tube was obstructed, necessitating replacement. Blind reinsertion was unsuccessful, prompting endoscopic assistance.

The patient was treated with piperacillin–tazobactam, ampicillin–sulbactam, fentanyl, propofol, furosemide, and omeprazole. Additionally, strong neominophagen C (glycyrrhizin) and norepinephrine were administered.

**Fig. 2:**
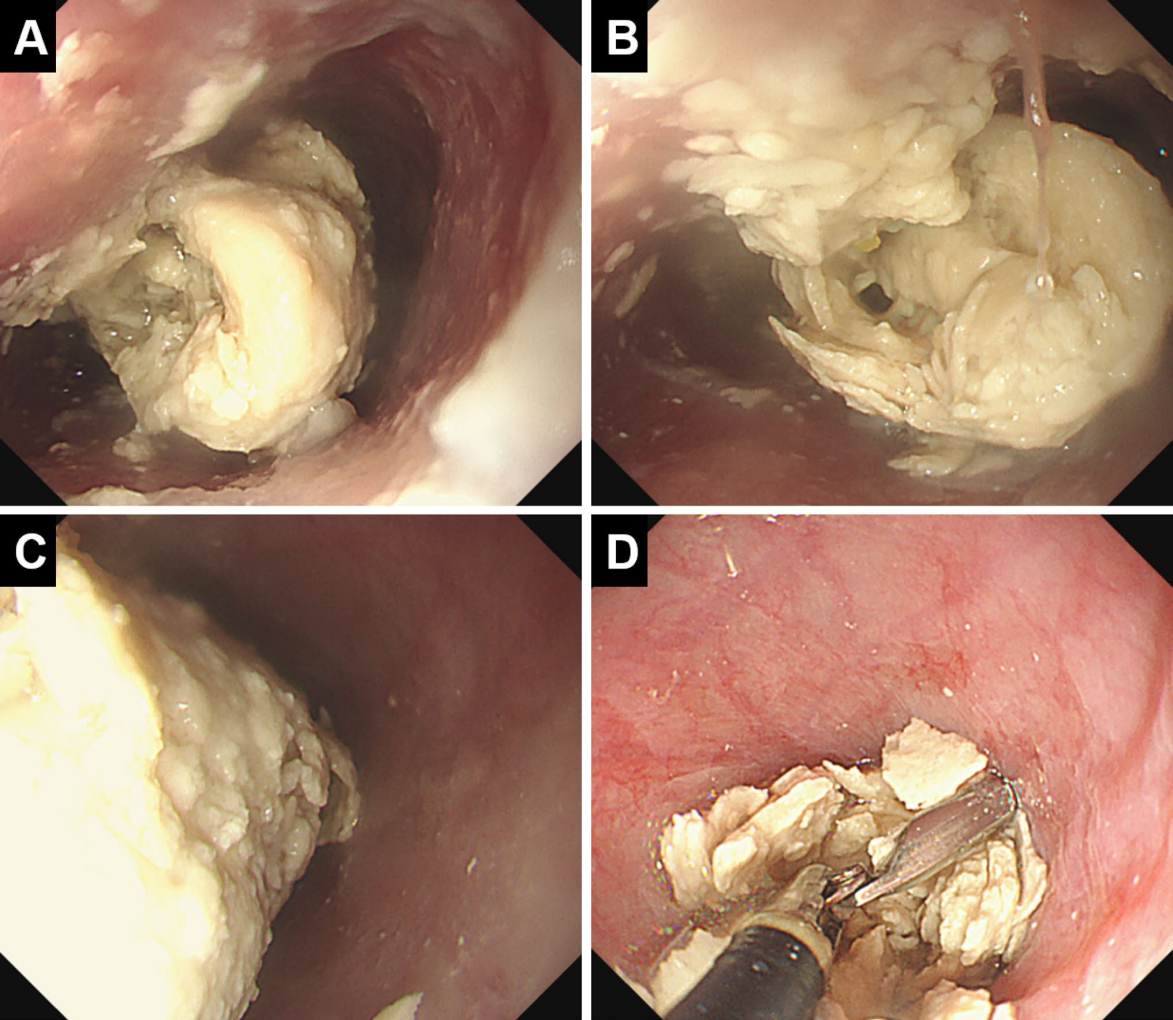
Endoscopic findings of the esophageal bezoar. Endoscopy reveals that the esophageal lumen is completely filled with white solid material (**A**–**C**). Repeated attempts at fragmentation using grasping forceps followed by suction are performed; however, the mass could not be sufficiently broken down for removal (**D**)

**Fig. 3:**
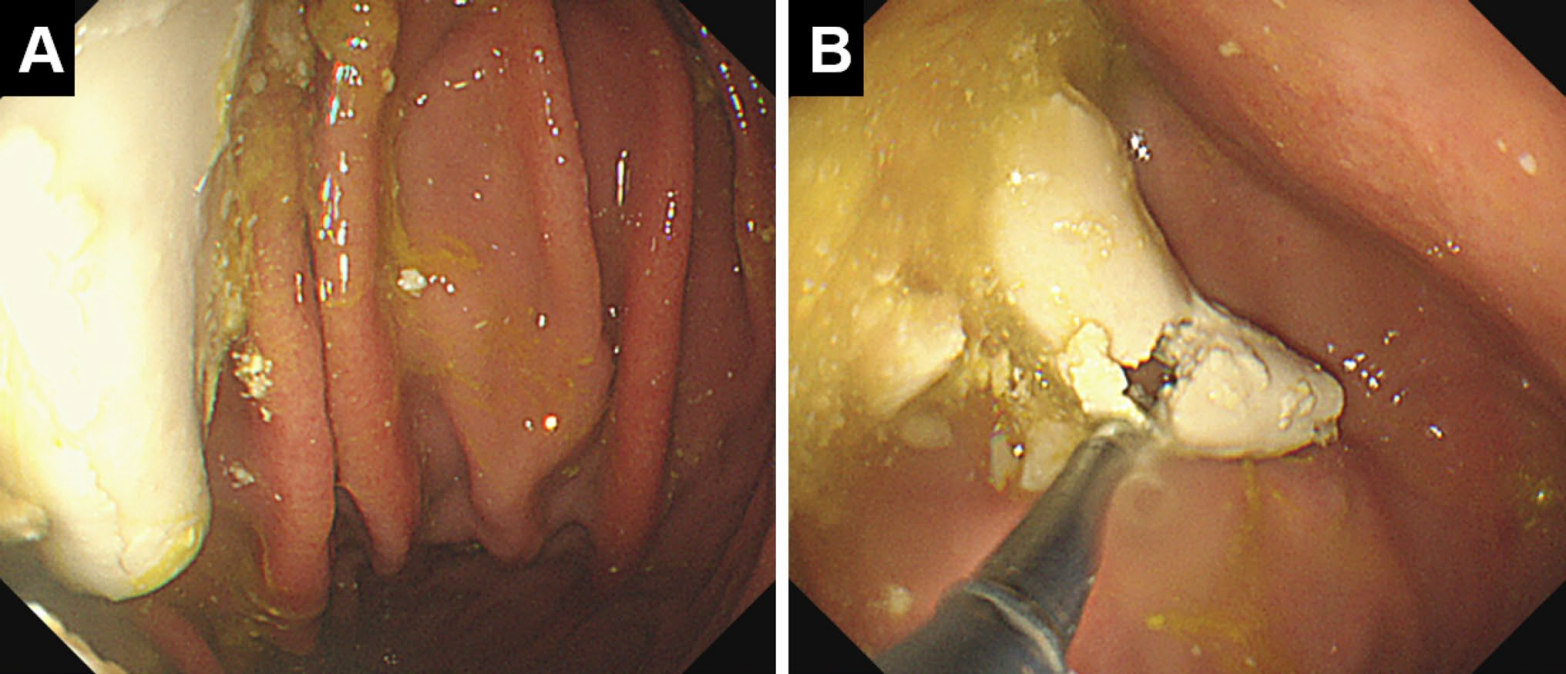
Endoscopic findings after dislodging the esophageal bezoar into the stomach. A large cylindrical white mass, molded to the shape of the esophageal lumen, is displaced into the stomach

Endoscopy revealed a white, solid material filling the esophageal lumen, obstructing nasogastric tube passage (Fig. 2). Initial fragmentation using biopsy forceps was insufficient to allow endoscope passage. Repeated fragmentation using grasping forceps, followed by suction, was performed; however, the mass could not be broken down sufficiently for removal. Ultimately, a large cylindrical white mass molded to the esophageal lumen’s shape was dislodged to the stomach (Fig. 3).

The esophageal obstruction was resolved, and a new nasogastric tube was successfully placed. The patient was successfully extubated and weaned from mechanical ventilation on the day after the endoscopic procedure. Enteral nutrition was resumed using Peptamen® (Nestlé Health Science, Switzerland). The postoperative course was uneventful with no esophageal bezoar recurrence, and the patient was discharged on POD 38.

## Experimental methods and results

An in vitro experiment was conducted to assess whether a casein-based enteral nutrition formula (Termeal® series) undergoes solidification under acidic conditions. Normal saline (Otsuka Pharmaceutical Co., Ltd., Japan) with an initial pH of 5.5 was used as the base solution. Hydrochloric acid (Nacalai Tesque, Inc., Japan) was added dropwise to prepare hydrochloric acid–adjusted saline solutions at pH 4.0, 3.0, 2.0, and 1.0. The pH 1.0 solution was defined as simulated gastric juice (hydrochloric acid–adjusted saline, pH 1.0, without pepsin).

**Fig. 4:**
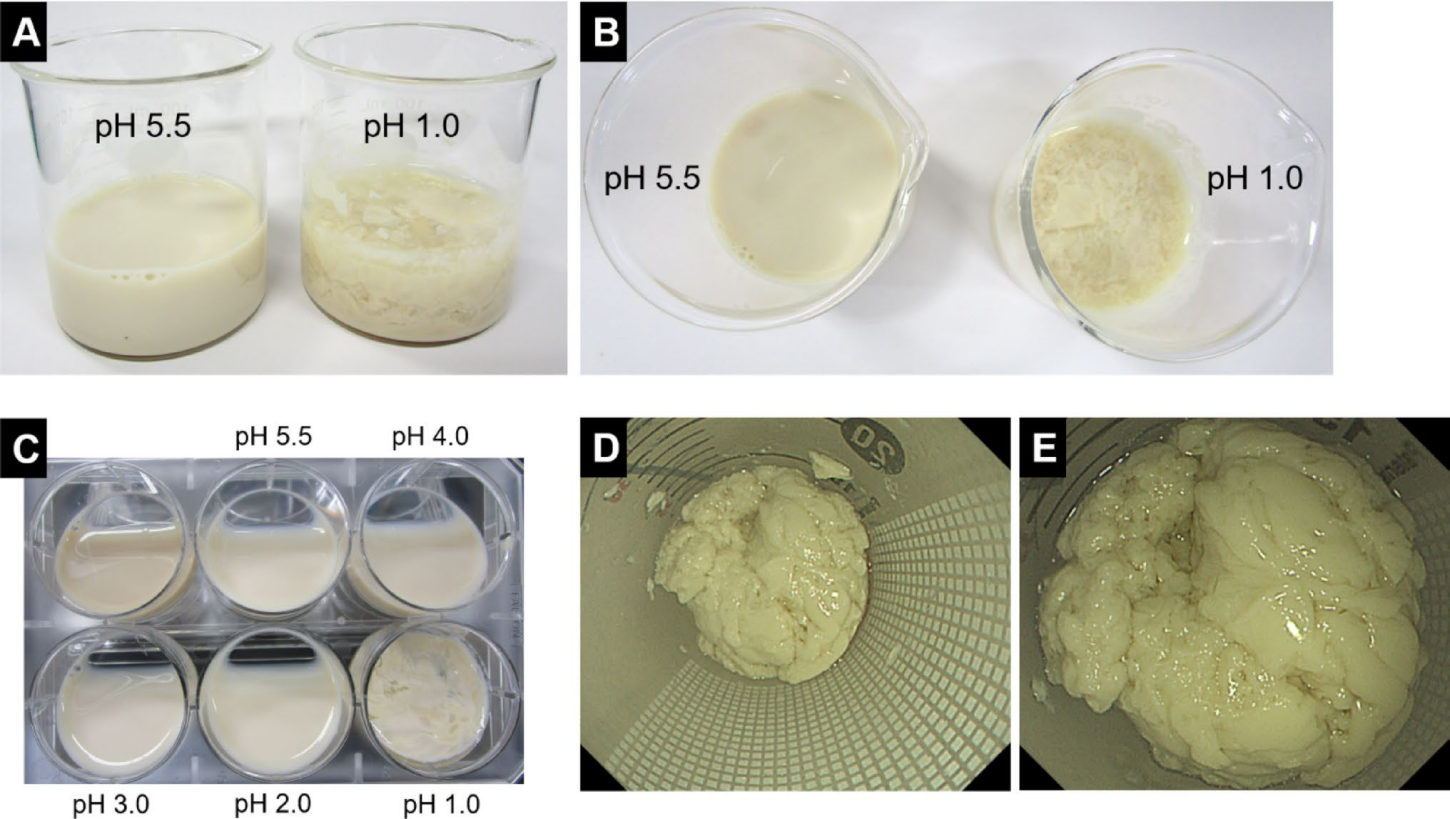
In vitro solidification of a casein-based enteral nutrition formula under acidic conditions. The casein-based enteral nutrition formula mixed at a 1:1 ratio with normal saline (pH 5.5) remained in a liquid state (**A**, **B**). A six-well plate experiment demonstrates the formula mixed 1:1 with hydrochloric acid–adjusted saline at pH 4.0, 3.0, 2.0, and simulated gastric juice (hydrochloric acid–adjusted saline, pH 1.0, without pepsin) (**C**). The mixtures remained liquid at pH 2.0–5.5, whereas rapid solidification was observed exclusively under the pH 1.0 condition. To objectively demonstrate the difference between the liquid and solid states, the plate was tilted during image acquisition. Endoscopic images show the solidified material obtained after mixing the casein-based formula with simulated gastric juice (pH 1.0) and allowing it to stand for 12 h in a conical tube (**D**, **E**)

First, 20 mL of the casein-based formula was mixed with 20 mL of normal saline in a glass beaker. The mixture remained liquid (Fig. 4A, B). In contrast, when 20 mL of the casein-based formula was mixed with 20 mL of simulated gastric juice, rapid solidification occurred.

Next, mixtures were prepared in a six-well plate as follows: 4 mL of the casein-based formula alone; and 2 mL of the formula mixed with 2 mL of hydrochloric acid–adjusted saline at pH 4.0, 3.0, 2.0, or simulated gastric juice (Fig. 4C). When the formula was mixed at a 1:1 ratio with solutions at pH 2.0–5.5, all mixtures remained liquid. In contrast, rapid and reproducible solidification was observed only in the mixture containing simulated gastric juice.

Finally, 20 mL of the casein-based formula was mixed with 20 mL of simulated gastric juice in a 50-mL conical tube and left undisturbed for 12 h. After removal of the liquid component, a solidified mass remained within the tube. Endoscopic images of the solidified material are shown in Fig. 4D and 4E.

## Discussion

Enteral nutrition-associated esophageal bezoars are rare but clinically significant complications, often linked to casein-containing formulas that solidify in acidic environments [3–7]. In this case, the enteral formula used (Termeal series) contained casein, consistent with previous studies. The in vitro experiment performed in this study provides mechanistic support for the formation of casein-based esophageal bezoars under highly acidic conditions. When the casein-based enteral formula was exposed to simulated gastric juice (hydrochloric acid–adjusted saline, pH 1.0, without pepsin), rapid and reproducible solidification occurred, whereas the formula remained liquid when mixed with solutions at pH 2.0–5.5. These findings suggest that extreme acidity alone, even in the absence of digestive enzymes such as pepsin, is sufficient to induce coagulation of casein-based formulas. This observation is consistent with the known physicochemical properties of casein micelles, which destabilize and aggregate at very low pH levels [8, 9], whereas peptide-based or whey-dominant products remain in solution, even under strong acidity.

It should be emphasized that this patient was receiving proton pump inhibitor therapy, making sustained exposure to strongly acidic gastric juice unlikely. However, intragastric pH under acid-suppressive therapy is not uniform and may fluctuate, particularly in the early postoperative period. Moreover, local physicochemical conditions within the esophagus, such as prolonged stasis of enteral formula, concentration due to dehydration, and limited clearance, may facilitate coagulation of casein-containing formulas even without continuous exposure to extreme acidity. Notably, our in vitro experiment demonstrated that solidification of the casein-based formula occurred rapidly upon exposure to a strongly acidic environment. This finding raises the possibility that even brief or transient exposure to localized acidic conditions, despite ongoing proton pump inhibitor therapy, could act as a trigger for coagulation of the enteral formula when it is retained within the esophagus.

Unlike cases involving elderly ICU patients with prolonged tube feeding and multiple comorbidities, our patient was a young postoperative individual who developed a bezoar within a relatively short duration of enteral nutrition. This highlights that even short-term administration of a casein-containing formula, if combined with postoperative esophageal stasis and reflux, can predispose patients to bezoar formation. Our findings suggest that the risk of bezoar development is not solely dependent on prolonged feeding duration or intrinsic esophageal motility disorders, but may also arise from brief episodes of postoperative stasis combined with exposure to highly acidic gastric juice. Consequently, careful selection of enteral formulas should be considered in postoperative or critically ill patients at risk of esophageal reflux and impaired clearance.

In addition to impaired esophageal peristalsis, nasogastric tubes may contribute to bezoar formation by promoting gastroesophageal reflux and facilitating pooling of enteral formula within the esophageal lumen [10, 11]. Several earlier reports have associated enteral formula bezoars with achalasia, systemic sclerosis, Guillain–Barré syndrome, and myasthenia gravis, all of which reduce esophageal clearance [12]. Our patient had no intrinsic motility disorder; however, prolonged supine positioning, repeated intubation, and postoperative dysphagia likely created sufficient stasis for casein-containing formula coagulation.

Medications commonly administered in intensive care settings, including opioids, sedatives, and certain antacids, may delay gastric emptying or contribute to bezoar formation [13]. Historical cases involved the use of sucralfate- or aluminum-based antacids, both of which have binding properties that facilitate concretion [4, 7, 12]. In our case, such agents were not administered; however, fentanyl and sedative medications were used for respiratory support, which may have diminished swallowing frequency and delayed esophageal clearance. Thus, although the medication profile differed from that of the earlier cases, the degree of pharmacological contribution could not be excluded.

Management strategies for casein-induced bezoars vary. Successful dissolution has been reported using pancreatic enzyme preparations, effervescent drinks, or prolonged lavage with bicarbonate-containing solutions [3, 4, 12]. Endoscopic fragmentation remains the most commonly employed method, although it is technically demanding and occasionally requires multiple sessions [12]. Rare cases of esophageal perforation after aggressive manipulation emphasize the need for caution [14]. Presently, the bezoar was only partially fragmented with forceps and suction; however, the degree of fragmentation was limited, and most of the mass was ultimately dislodged into the stomach for removal.

Preventive measures include semi-recumbent feeding to reduce reflux, routine flushing of feeding tubes, acid-suppressive therapy, prokinetics, and the selection of non-casein formulas for high-risk patients [3, 4]. Potassium-competitive acid blockers (P-CABs) provide more rapid and potent gastric acid suppression than conventional proton pump inhibitors and may more promptly elevate intragastric pH. From a theoretical standpoint, administration of a P-CAB via a nasogastric tube during enteral feeding with casein-based formulas could reduce the likelihood of casein coagulation by minimizing exposure to highly acidic conditions. Although the preventive efficacy of P-CABs for enteral nutrition–associated bezoars has not been systematically evaluated, such an approach may be considered as a potential preventive strategy in postoperative or critically ill patients at high risk for esophageal stasis and reflux. Nasointestinal feeding routes may decrease esophageal exposure to enteral formulas.

## Conclusion

This case demonstrates that esophageal bezoars can develop even during short-term postoperative enteral nutrition when casein-containing formulas are administered under conditions of impaired esophageal clearance. The accompanying in vitro findings suggest that brief exposure of casein-based formulas to a highly acidic environment may induce rapid solidification, representing one of several plausible mechanisms contributing to bezoar formation in this patient. Clinicians should be aware of this potential complication when encountering feeding intolerance or nasogastric tube obstruction during enteral nutrition with casein-containing formulas.
